# Optimization of Phytochemical-Rich *Citrus maxima* Albedo Extract Using Response Surface Methodology

**DOI:** 10.3390/molecules28104121

**Published:** 2023-05-16

**Authors:** Woorawee Inthachat, Piya Temviriyanukul, Nattira On-Nom, Panyaporn Kanoongon, Sirinapa Thangsiri, Chaowanee Chupeerach, Uthaiwan Suttisansanee

**Affiliations:** Food and Nutrition Academic and Research Cluster, Institute of Nutrition, Mahidol University, Salaya, Phuttamonthon, Nakhon Pathom 73170, Thailand; woorawee.int@mahidol.ac.th (W.I.); nattira.onn@mahidol.ac.th (N.O.-N.); pang_kanoongon@hotmail.com (P.K.); sirinapa.tha@mahidol.ac.th (S.T.); chaowanee.chu@mahidol.ac.th (C.C.)

**Keywords:** agricultural waste, antioxidant activities, *Citrus maxima*, enzyme inhibition, flavonoids, genotoxicity, naringenin, phenolics, response surface methodology, sustainability

## Abstract

In the present study, response surface methodology (RSM) and Box–Behnken design (BBD) were employed to optimize the conditions for the extraction of *C. maxima* albedo from agricultural waste, to obtain notable phytochemicals. Ethanol concentration, extraction temperature, and extraction time were included as key factors contributing to the extraction. The results showed that the optimum extraction condition for *C. maxima* albedo was 50% (*v*/*v*) aqueous ethanol at 30 °C for 4 h, which provided total phenolic contents and total flavonoid contents at 15.79 mg of gallic equivalent/g dry weight (DW) and 4.50 mg of quercetin equivalent/g DW, respectively. Considerable amounts of hesperidin and naringenin at 161.03 and 3430.41 µg/g DW, respectively, were detected in the optimized extract using liquid chromatography–electrospray ionization–tandem mass spectrometry (LC–ESI–MS/MS). The extract was later subjected to a test for its enzyme-inhibitory activities against key enzymes relevant to Alzheimer’s disease (AD), obesity, and diabetes as well as for its mutagenicity potential. Among enzyme inhibitory activities, the extract showed the highest inhibitory strength against β-secretase (BACE-1), which is a drug target for AD treatment. The extract was also devoid of mutagenicity properties. Overall, this study demonstrated a simple and optimal extraction procedure for *C. maxima* albedo with a significant quantity of phytochemicals, health benefits, and genome safety.

## 1. Introduction

*Citrus maxima* (Burm.) Merr. (*C. maxima*) or pomelo in the Rutaceae family is widely known as the largest citrus in the citrus family. *C. maxima* is a medium-sized perennial plant with a 6–8 m trunk. Its fruit has a diameter of 12–18 cm, while the fruit wall is 2–2.5 cm thick. A green exocarp known as flavedo is the oil-rich part with a distinctive aroma and is frequently employed in the extraction of essential oils [1]. A white spongy mesocarp known as albedo is approximately 2 cm thick, depending on cultivars [1]. This part is rarely ingested due to its bitter taste, thereby becoming agricultural waste. Overall, the citrus-processing industries are involved in the mass production of waste, accounting for over 50–60% of the mass of fresh fruit [2]. Hence, poor management of citrus waste can have severe negative environmental impacts due to its high organic-acid content. Fortunately, it was previously reported that citrus peel contains large quantities of phytochemicals, including phenolic acids and flavonoids, which are well-known for their health-promoting bioactivities as antioxidants, anti-obesity agents, anti-diabetics, etc. [3]. The value of these remaining phytochemicals could be increased in the nutraceutical and functional food sectors. Therefore, extraction of phytochemicals from *C. maxima* peel may be a sustainable way to reduce food waste and promote agricultural waste value.

It was previously shown that the concentration of ethanol (as the extraction solvent), pH of the solvent, and extraction temperature greatly impacted the extraction yields of flavonoids and limonoids, such as hesperidin and limonin, respectively, in lime (*C. aurantifolia*) peel [4]. The extraction yield of hesperidin was increased when the ethanol concentration (as percentage of ethanol in water) was increased in aqueous ethanolic solvents ranging from 60–80% [4]. However, the extraction yield declined in absolute ethanol [4]. A similar trend was observed in *C. limon* cv. Meyer peel, in which total phenolic contents gradually increased with 20–72% (*v*/*v*) aqueous ethanol, and declined when reaching 95% (*v*/*v*) aqueous ethanol [5]. However, an elevated extraction temperature seemed to decrease the extraction efficacy of flavonoids and limonoids in lime peel [4], while increasing phenolic contents in *C. limon* cv. Meyer peel [5]. In addition, it was found that extraction time could also affect the extraction efficacy of phytochemicals in *C. maxima* peel using microwave-assisted extraction (MWE) [6]. Interestingly, the phytochemicals in *C. maxima* peel remained unchanged under different extraction temperatures in an ultrasound-assisted extraction (UAE) [6]. Therefore, three main factors including ethanol concentration, extraction temperature, and extraction time were selected in analysis to optimize the extraction conditions of *C. maxima* albedo to obtain a high quantity of phytochemicals. Moreover, response surface methodology (RSM) and Box–Behnken design (BBD) were used to design the extraction conditions for this study. RSM uses mathematical and statistical principles to analyze the correlation of investigated parameters under optimum conditions. BBD is used to analyze the number of trials, leading to a reduced likelihood of complications, in a time-saving, cost-effective, and full factorial approach which reduces the complexity of analysis while maintaining a high degree of accuracy [7].

Overall, the purpose of this study was to optimize the extraction conditions of *C. maxima* albedo in order to acquire a high quantity of phytochemical contents using a simple extraction procedure. The health-promoting bioactivities of the obtained extracts were also investigated for their antioxidant activities and enzyme-inhibitory activities against key enzymes involved in some non-communicable diseases (NCDs), including diabetes (α-amylase and α-glucosidase), obesity (lipase), and Alzheimer’s disease (acetylcholinesterase, butyrylcholinesterase, and β-secretase). In addition, volatile compounds and genotoxicity were also determined by using the bacterial reverse mutation test (Ames test) in line with the recommendations of the Organization for Economic Cooperation and Development (OECD) [8]. The knowledge gained from this research is an excellent example of increasing the usefulness and value of the extract from agricultural waste.

## 2. Results

### 2.1. Optimization of Extraction Conditions Using Response Surface Methodology

#### 2.1.1. Model Fitting, Analysis of Variance, and Validation

As previously mentioned, ethanol concentration, extraction temperature, and extraction time play a role in the extraction efficacy to obtain high amounts of phytochemicals. Hence, these three factors were selected as independent variables at three levels (−1 to 1) in the BBD. A total of 30 randomized runs derived from BBD, together with total phenolic contents (TPCs) and total flavonoid contents (TFCs) as dependent variables, were designed (Table 1). The coded and uncoded variables were selected as X_1_: ethanol concentration (50–90% (*v*/*v*) aqueous ethanol), X_2_: extraction temperature (30–70 °C), and X_3_: extraction time (2–6 h). The TPCs of *C. maxima* albedo extract were in the range of 4.47–21.88 mg gallic acid equivalent (GAE)/g dry weight (DW) with the highest being observed in X_1_:X_2_:X_3_ = 70% *v*/*v*:70 °C:6 h. The TFCs ranged 1.40–4.50 mg quercetin equivalent (QE)/g DW with the highest being observed in X_1_:X_2_:X_3_ = 50% *v*/*v*:30 °C:4 h. These data imply that the effectiveness of the extraction depends on the employed conditions.

**Figure 1:**
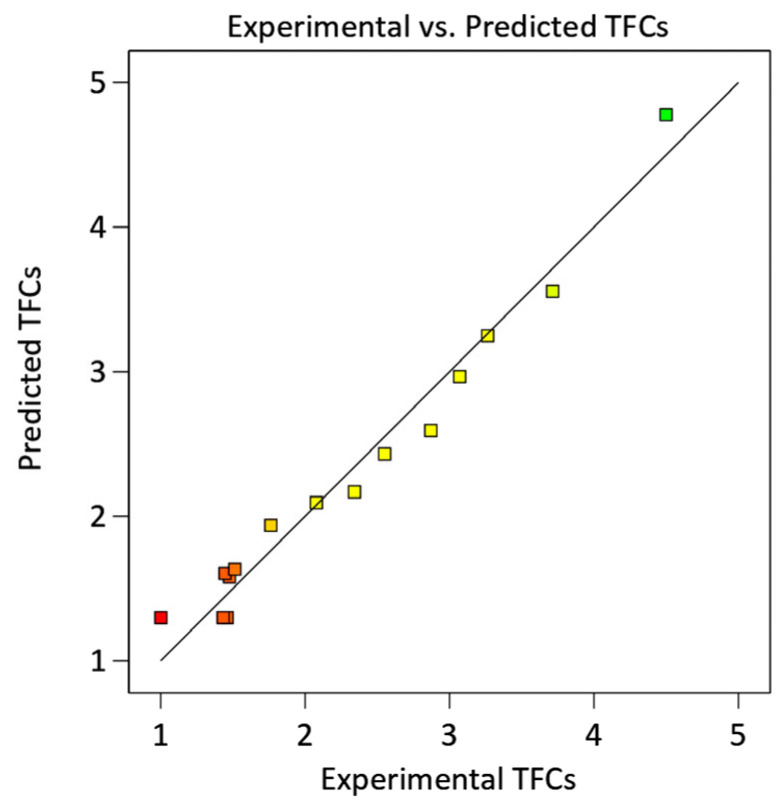
Experimentally measured total flavonoid contents (TFCs) vs. predicted TFCs calculated from Equation (1).

The second-order polynomial equations were selected for further establishing the optimal extraction conditions of *C. maxima* albedo regarding its TPCs and TFCs. The coefficient of determination (R^2^), regression coefficients, lack of fit, and *p*-values of the second-order polynomial models for TPCs and TFCs are shown in Table 2. Unfortunately, the analysis of variance (ANOVA) data indicated that the three independent variables (ethanol concentration, extraction temperature, and extraction time) were uncorrelated to the TPC data as the model was statistically insignificant (*p* = 0.4440) with the *p*-value of lack of fit lower than 0.05 (*p* < 0.0001). In addition, the R^2^ was only 0.5923, indicating a weak relationship between experimental and predicted responses. Intriguingly, for TFCs, the model was statistically significant (*p* = 0.0030). In addition, the *p*-value of the monomial coefficient, $X_{1},$ was less than 0.01, indicating that ethanol concentration had a linear relationship and was crucial for TFCs extraction, while the *p*-values of the monomial coefficients, $X_{2}$ and $X_{3},$ were all greater than 0.05, indicating that the amount of TFCs was affected by neither extraction temperature nor extraction time. Furthermore, the *p*-value of the interaction coefficient of $X_{1}X_{2}$ was lower than 0.05 (*p* = 0.0195), indicating that this pairwise interaction model of ethanol concentration and extraction temperature was highly significant for TFC extraction. The quadratic coefficients, $X_{1}^{2}$ and $X_{2}^{2},$ were also significant (*p* < 0.001 and *p* < 0.01, respectively), confirming that the TFCs were significantly influenced by the quadratic factors of ethanol concentration and extraction temperature. The other two interaction coefficients, $X_{1}X_{3}$ and $X_{2}X_{3},$ were insignificant (*p* > 0.05), indicating that neither the interaction between ethanol concentration and extraction time nor between extraction temperature and extraction time impacted the extraction of flavonoids from *C. maxima* albedo. The R^2^ and its adjusted values (R^2^ adjusted) for TFCs were 0.9686 and 0.9122, respectively, which were in close proximity to 1.0, indicating that the model possessed a high degree of validity in predicting TFCs. Finally, the predicted second-order polynomial regression equations for TFCs were generated, as shown in the Equation (1).

(1) $$Y=1.30-0.834X_{1}-0.257X_{2}-\;0.142X_{3}+0.507X_{1}X_{2}+0.153X_{1}X_{3}-\;0.010X_{2}X_{3}+1.130X_{1}^{2}+0.748X_{2}^{2}-0.004X_{3}^{2}$$

where Y is the predicted TFCs (mg QE/g DW) and $X_{1},{\;X}_{2},{\;\mathrm{and}\;X}_{3}$ are the independent variables including ethanol concentration (% *v*/*v*), extraction temperature (°C), and extraction time (h), respectively. The prediction equation (Equation (1)) was used to calculate the TFCs and compared with the experimental data, as shown in Figure 1. The data showed that the TFCs obtained from experiment and prediction were aligned with a high degree of correlation, confirming the validity of the Equation (1).

#### 2.1.2. The Effect of Extraction Conditions on Total Flavonoid Contents

**Figure 2:**
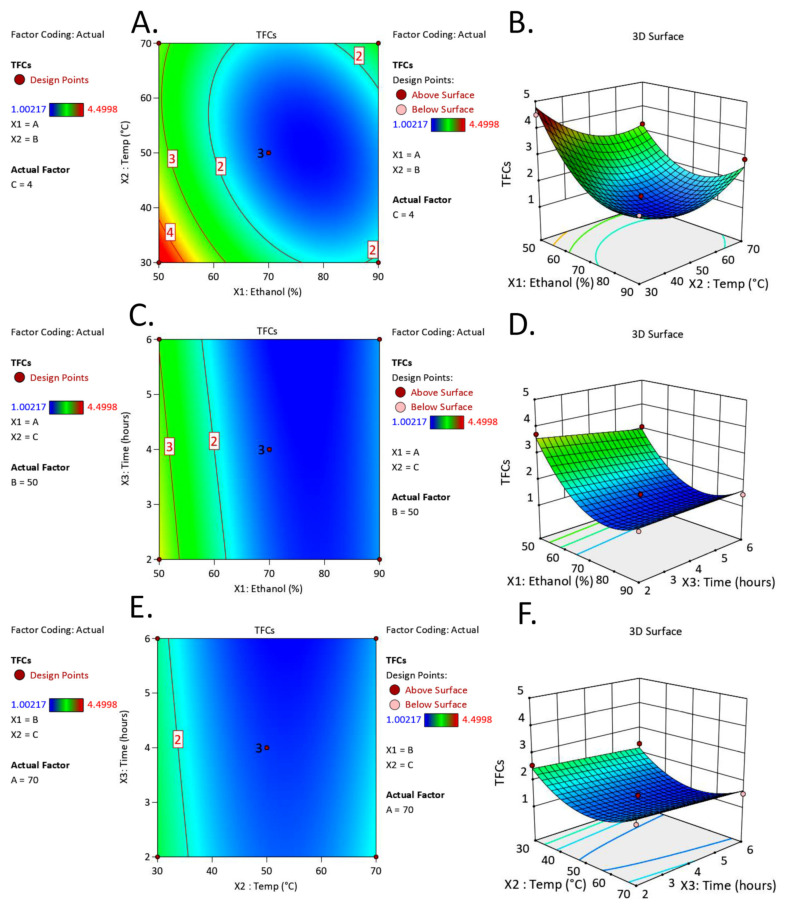
Contour plots (**A**,**C**,**E**) and response surface plots (**B**,**D**,**F**) of TFCs affected by ethanol concentration (X_1_), extraction temperature (X_2_), and extraction time (X_3_).

The complexity and role of each factor in TFC extraction were further studied using RSM analysis. The contour and three-dimensional (3D) response surface plots provided details on the strength of the interactions between independent variables and demonstrated the mutual influence of any two independent variables on the dependent variables (Figure 2). The interactions were investigated between two variables of $X_{1}X_{2}$, $X_{1}X_{3}$, and $X_{2}X_{3}$, where $X_{1}$ is ethanol concentration, $X_{2}$ is extraction temperature, and $X_{3}$ is extraction time on the response variable, TFCs. The contour and response surface plots of $X_{1}X_{2}$ (Figure 2A,B) show the influences of ethanol concentration and extraction temperature on the extraction of TFCs, indicating that increasing temperature in the extraction process resulted in increased TFCs. However, increasing temperature to more than 40 °C could yield low TFC extraction efficacy. The same pattern was observed in ethanol concentration. Gradually increased ethanol concentration could elevate TFCs; however, TFCs started to decline with more than 55% (*v*/*v*) aqueous ethanol. The interactions between variables of $X_{1}X_{3}$ also suggested that an initial increase in ethanol concentration (50–70%) led to decreased TFCs. However, the expansion in the extraction processing time from 2 to 6 h had no effect on the TFCs (Figure 2C,D). Furthermore, the relationship between the extraction time and extraction temperature ($X_{2}X_{3}$) showed that extraction processes with different time intervals of 2–6 h at the particular temperature had minor effects on the TFCs (Figure 2E,F). However, the initial increase of the extraction temperature led to an increase in TFCs and was followed by a decline after a particular point of extraction temperature. Thus, it is clear that an appropriate ethanol concentration and extraction temperature (only with the combination of ethanol) contributes to the extraction efficacy for TFCs in *C. maxima* albedo, while extraction time had no effect.

The optimized extraction conditions determined by Design-Expert were 50.92% (*v*/*v*) aqueous ethanol, 30.10 °C for the temperature, and 4.30 h extraction time. The expected TFC in this condition was 4.56 mg QE/g DW. Nevertheless, for reproducibility purposes, we adjusted the conditions to 50% (*v*/*v*) aqueous ethanol, 30 °C extraction temperature, and 4 h extraction time. Under these adjusted conditions, the TFC was found to be 4.50 ± 0.01 mg QE/g DW. In addition, it was found that *C. maxima* albedo extract also exhibited a TPC of 15.79 ± 0.03 mg GAE/g DW, even though this model is not correlated to TPCs.

### 2.2. Analysis of Phytochemical Profile

**Figure 3:**
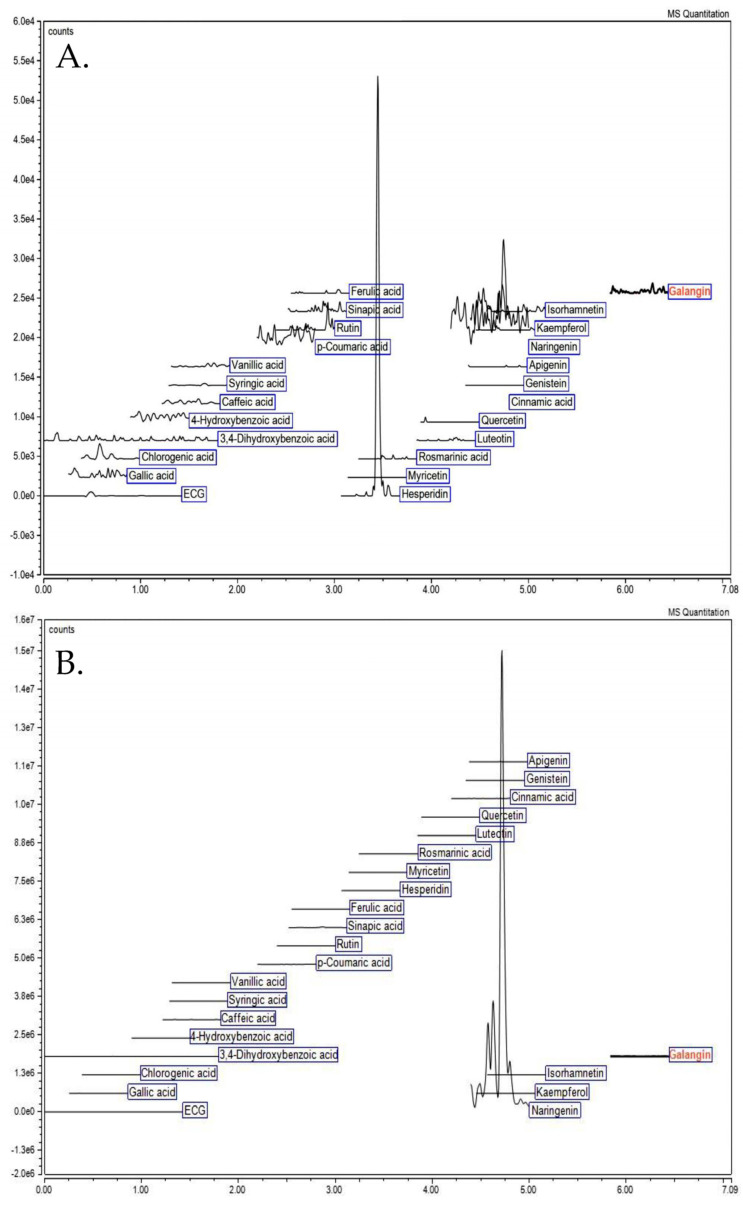
The liquid chromatography–electrospray ionization–tandem mass spectrometry (LC–ESI–MS/MS) chromatogram of *C. maxima* albedo extracted under optimized extraction conditions. (**A**) under non-hydrolysis conditions and (**B**) under acid hydrolysis conditions.

Under optimized extraction conditions in Section 2.1, the phytochemical profile of *C. maxima* albedo extract was investigated using liquid chromatography–electrospray ionization–tandem mass spectrometry (LC–ESI–MS/MS), as shown in Figure 3 and Table 3.

Under non-acid hydrolysis, the *C. maxima* albedo extract contained a single flavonoid, hesperidin, at the concentration of 161.03 µg/g DW. However, naringenin was predominantly detected (3430.41 µg/g DW) under acid hydrolysis, while lower amounts of sinapic acid and apigenin were also observed (24.1- and 81.8-fold lower, respectively).

### 2.3. Analysis of Volatile Compounds

**Figure 4:**
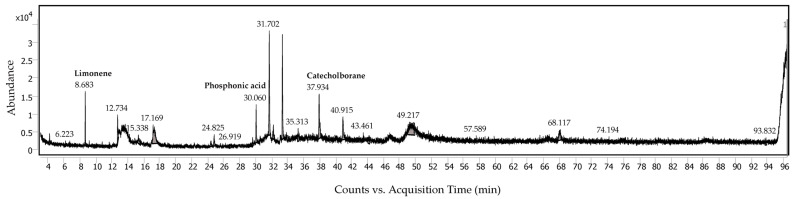
The gas chromatography–mass spectrometry (GC–MS) chromatogram of *C. maxima* albedo extract using a liquid-mode analysis.

Utilizing gas chromatography–mass spectrometry (GC–MS) analysis, volatile compounds of *C. maxima* albedo were characterized since they were previously reported to be abundant in the citrus peel [9]. According to the GC–MS data (Figure 4 and Table 4), more than 27 peaks were recorded; however, only 3 peaks attributable to limonene, 4-hydroxybenzenephosphonic acid and catecholborane were identified from the National Institute of Standards and Technology (NIST) database using the possibility score of more than 50%. Limonene is a cyclic monoterpene found primarily in the citrus peel. It is still unclear for the presence of phosphonic acid, (phydroxyphenyl)- (or 4-hydroxybenzenephosphonic acid) and catecholborane. Further elucidation requires further investigation.

### 2.4. TPCs, TFCs, Antioxidant Activities, and Enzyme Inhibitory Activities

Flavonoids have been widely documented for their health-promoting activities [10] and are usually utilized in the functional food sectors. Hence, to promote and enhance the usefulness of the extract requires the antioxidant and health-promoting bioactivities of *C. maxima* albedo extracted under optimized extraction conditions (Table 5). The antioxidant activities were determined by DPPH radical scavenging, FRAP, and ORAC assays. The first two follow the single electron transfer (SET) mechanism, while the last follows the hydrogen atom transfer (HAT) mechanism. The results indicated that high ORAC activities were detected at 675.34 µmol Trolox equivalent (TE)/g DW, while lower activities at 15.35 and 9.36 µmol TE/g DW were detected by the DPPH radical scavenging and FRAP assays, respectively.

Interestingly, the extract exhibited enzyme inhibitory activities against all tested enzymes, albeit at different degrees of inhibition. The highest enzyme inhibitory activity was observed in β-secretase (BACE-1) (54.61% inhibition at the extraction concentration of 8 mg/mL), the β-amyloid formation enzyme, which is a drug target for Alzheimer’s disease (AD) treatment. Thus, inhibition of BACE-1 could lead to reducing risk of AD occurrence. Anti-AD properties through controlling of neurotransmitter degrading enzymes, acetylcholinesterase (AChE) and butyrylcholinesterase (BChE), were also determined. However, the inhibitory activities were much lower compared to BACE-1. This suggests that the extract possesses anti-AD properties by inhibiting the amyloid pathway rather than the cholinergic pathway. In addition to anti-AD properties, the extract (the final extraction concentration of 25 mg/mL) seemed to exhibit anti-diabetic properties through the inhibition of α-glucosidase (45.40% inhibition) and α-amylase (35.38% inhibition), while minor effects on anti-obesity were observed in lipase inhibition at 20.89% inhibition. In summary, *C. maxima* albedo extracted under optimized extraction conditions possesses potential antioxidant activities, probably via the HAT mechanism, and anti-AD (through the amyloidogenic pathway) and anti-diabetic activities.

### 2.5. Genotoxicity Testing Using Bacterial Reverse Mutation Test (Ames Test)

One of the requirements for functional food development is genotoxicity testing; thus, the genotoxicity potential of the *C. maxima* albedo extract was evaluated using the Ames test in line with the recommendation of the Organization for Economic Co-operation and Development (OECD 471) [8]. Five *S. typhimurium* strains including TA98, TA100, TA102, TA1535, and TA1537 were employed to cover various types of mutations, such as frameshift and missense (base substituted) mutations [11]. The number of revertant colonies of each *S. typhimurium* strain were compared with the recommended positive control without bioactivation from liver extract (−S9) (Table 6), while the number of revertant colonies of each *S. typhimurium* strain were compared with the recommended positive control with bioactivation from liver extract (+S9) (Table 7). The liver extract was added to the assay to evaluate whether the *C. maxima* albedo extract could act as a direct- or indirect-acting mutagen. No increases in the number of revertant colonies were detected in any of the five *S. typhimurium* strains in the absence or in the presence of S9 extract. The mutagenicity ratios (MRs) were also similar to those of a negative control (a solvent control of DMSO) whereas all positive controls including 4-nitroquinoline-1-oxide (4-NQO), sodium azide (NaN_3_), mitomycin C (MMC), 9-aminoacridine (9-AA), and 2-aminoanthracen (2-AA) showed significant increases in the revertant colonies compared with a negative control, leading to high MR. Thus, it was clearly shown that the *C. maxima* albedo extract, up to 2000 µg/plate, did not induce DNA mutations.

## 3. Discussion

Pomelo (*C. maxima*) is frequently utilized in the citrus-juice industry because it is dense in nutrients and vitamins [12]. However, a huge accumulation of food waste is formed as approximately just 40% of the total fruit can be used, while the remaining 50–60% comprising peel, pomace, and seeds is discarded [2]. This organic waste eventually impacts the environment as it may contribute to soil acidity [2]. It has been previously documented that citrus peels, including flavedo and albedo, are rich in volatile compounds and phytochemicals, which are meaningful in the nutraceutical industries [3]. Flavedo is a common subject for essential oil extraction, leaving the albedo as a real food waste with no value. Thus, obtaining phytochemicals that remain in the albedo part could be one alternative way to reduce the negative environmental impact while adding value to the citrus albedo. In this study, we employed a multivariate strategy (BBD) together with RSM analysis to optimize the extraction conditions of *C. maxima* albedo and achieve high amounts of phytochemicals with simple extraction procedures. The extract obtained from the optimized extraction conditions was later subjected to analysis of phytochemical profiles, health-promoting activities, and genotoxicity.

In the present study, the three main factors affecting extraction efficacy were ethanol concentration, extraction temperature, and extraction time. Ethanol was chosen due to its low toxicity compared to methanol [13] and its environmental friendliness, low cost, and general acceptance in the nutraceutical and food industries [14]. According to ANOVA data (Table 2), ethanol was the only factor contributing to the extraction efficacy of *C. maxima* albedo, while extraction temperature and time had a negligible effect. Although extraction temperature can be neglected as a single factor, it seemed to contribute to extraction efficacy when ethanol was used as the extraction solvent (Table 2). Our result corresponded to the previous report, indicating that ethanol and extraction temperature affected the yield of TFCs of *Citrus grandis* (L.) Osbeck (synonym of *C. maxima*) [15]. However, it was also found that an increased ethanol concentration led to a significant reduction of both TPCs and TFCs in *C. maxima* and *C. aurantifolia* [4,15]. These results were in line with our study as illustrated by the contour plot (Figure 2A). Aqueous ethanol is one of the common solvents for flavonoid extraction [16]. Our study indicated that 50% (*v*/*v*) aqueous ethanol (polarity index of 7.1 [17]) may be suitable for the extraction of flavonoids from *C. maxima* albedo, suggesting (i) flavonoids present in this plant part contain a similar polarity index to that of 50% (*v*/*v*) aqueous ethanol or (ii) a high water percentage in the extraction solvent may help plant cell swelling, resulting in high penetration of ethanol [18]. It is perplexing that extraction temperature influenced extraction efficacy only when ethanol was employed (Table 2), and a high temperature (higher than 40 °C) reduced the TFCs (Figure 2A). Temperature increased the extraction efficacy, possibly through increased solubility. This observation could be due to the gradually increased extraction temperature that may result in the biodegradation of phytochemicals. For instance, limonin and hesperidin, phytonutrients found in citrus, are thermally sensitive [19,20]. Furthermore, heat can mediate the conversion of phytochemicals into their derivatives [21], which might not be detected by simple TPC and TFC spectrophotometry, leading to the reduction of both TPCs and TFCs under the application of a high extraction temperature.

The present results showed that the optimal condition for *C. maxima* albedo was at 50% (*v*/*v*) aqueous ethanol, extraction temperature of 30 °C, and extraction time of 4 h. This optimal condition was well-matched with the flavonoid prediction rather than that of the phenolics (Table 2). Although there is no previous report on TFCs, it was found that the TPCs of *C. grandis* albedo from eight cultivars planted in Vietnam and Thailand ranged between 1.18 and 5.30 mg GAE/g DW [22,23]. Our result for TPCs (15.79 mg GAE/g DW) was higher than those of these previously reported TPCs, which might have been due to both external (such as growth environment, harvesting time, and extraction methods) and internal (such as cultivar and maturity stage) factors.

Moreover, the main flavonoids commonly present in citrus, including hesperidin (161.03 µg/g DW) and naringenin (3430.41 µg/g DW), were detected by LC–ESI–MS/MS. It is interesting that our extraction procedure could recover hesperidin since several attempts (ultrasound-assisted extraction (UAE) and conventional extraction using methanol and DMSO as the extraction solvent) failed to extract hesperidin from *C. maxima* peel [24,25]. The evidence of naringenin content in *C. maxima* peel is limited, and most studies have focused on naringin. Since naringin is poorly absorbed compared to naringenin (naringin aglycone), rendering it low in health-promoting properties [26], we, therefore, paid attention to naringenin. Several reports have shown that the naringenin contents in the peels of other citrus types were lower than in our present study. For example, *C. sinensis* peels (a hybrid between *C. maxima* and *C. reticulata*) extracted using various methods showed naringenin in concentrations in the range of 4–112 µg/g DW [27], while peels of fourteen citrus cultivars extracted by UAE showed naringenin contents of 30–260 µg/g DW [28]. Together with the TPCs and TFCs, the present study shows the advantages of BBD and RSM in the optimization of extraction to obtain high amounts of phytochemicals from the food waste of *C. maxima* albedo.

To enhance the utility of *C. maxima* albedo extract, the antioxidant and enzyme inhibitory activities against the key enzymes involved in NCDs were studied. Three antioxidant assays were employed, although the ORAC assay is a biologically relevant assay compared with DPPH radical scavenging and FRAP assays [29]. Distinctly high ORAC activities are seen in *C. maxima* albedo extract, implying that it could inhibit free radicals via the HAT mechanism instead of the SET mechanism as determined by DPPH radical scavenging and FRAP assays [30]. Amounts of TPCs and TFCs are commonly related to these properties, as reported by several studies [22,31,32]. In addition, *C. maxima* albedo extract exhibited wide ranges of inhibitory properties against enzymes, which are drug targets for the treatment of AD (AChE, BChE, and BACE-1), obesity (lipase), and diabetes (α-glucosidase and α-amylase). Interestingly, the obtained extract predominantly exhibited a strong inhibitory effect against BACE-1, which is an enzyme involved in AD pathogenesis. BACE-1 cleaves amyloid precursor protein (APP), eventually resulting in the formation of cytotoxic amyloid peptides, which are a hallmark of AD [33]. Hence, BACE-1 inhibitors could be developed to cure AD. Naringenin and hesperidin were the predominant flavonoids in *C. maxima* albedo extract (Table 5). Hesperidin had been reported to decrease BACE-1 activities and amyloid peptides in both the hippocampus and cortex in aluminum-chloride-induced AD in rats [34]. Naringenin had also been reported for its property as a BACE-1 inhibitor, leading to a reduction in amyloid peptides in high-fat-diet-fed mice [35]. Hesperidin and naringenin have a half maximal inhibitory concentration (IC_50_) against BACE-1 of 16.9 and 30.3 µM, respectively [36], suggesting the potential BACE-1 inhibitory activities of these two compounds found in our extract. However, since we determined the anti-AD using in vitro assay, the extract was not hydrolyzed by acid conditions, thereby only hesperidin was mainly considered. Hesperidin in our extract was 2.11 µM in 8 mg/mL extract, indicating that hesperidin may play a part as BACE-1 inhibitor and other compounds may contribute to the inhibitory activities. We guessed that naringin might be that compound because naringenin was predominant in the extract after acid hydrolysis. Naringin, a flavanone-7-*O*-glycoside, is formed between disaccharide and naringenin, and the disaccharide will be removed in acid conditions, resulting in naringenin detected, as shown in Table 5. Among 119 tested compounds, naringin was one of the promising compounds as an AChE, BChE, and BACE-1 blocker because, in the molecular docking study, naringin exhibited high binding affinity relative to donepezil and elenbecestat, the AD drugs [37].

Although this study developed the extraction condition of *C. maxima* albedo with a simple extraction procedure to achieve high phytochemical content and characterized its health-promoting activities and genotoxicity, antinutrient compounds, such as oxalate, phytic acid, and tannin, which are abundant in the *C. maxima* peel, as previously reported [12], were not determined. In addition, for functional food development, genotoxicity determination is one of the safety aspects; thus, sub-chronic or chronic toxicity testing is worth investigating.

## 4. Materials and Methods

### 4.1. Chemicals and Reagents

All chemicals used were of analytical grade. Sodium nitrite (NaNO_2_), aluminum chloride (AlCl_3_), sodium hydroxide (NaOH), Folin & Ciocalteu’s phenol reagent, sodium carbonate (Na_2_CO_3_), 2,3,5-triphenyltetrazolium chloride (TPTZ), 2,2-diphenyl-1-picrylhydrazyl (DPPH), iron (III) chloride (FeCl_3_), and S9 from rat liver were purchased from Sigma-Aldrich (St. Louis, MO, USA). 2,2′-Azobis-2-methylpropanimidamide dihydrochloride (AAPH) was purchased from Cayman Chemical (Ann Arbor, MI, USA). Reagent standards including gallic acid hydrate, quercetin hydrate, and 6-hydroxy-2,5,7,8-tetramethylchroman-2-carboxylic acid (Trolox) were purchased from Tokyo Chemical Industry Co., Ltd. (Tokyo, Japan).

### 4.2. Sample Selection, Preparation, and Extraction

**Figure 5:**
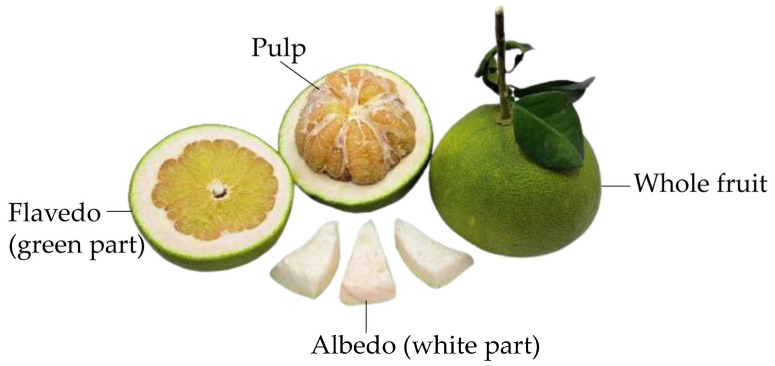
An illustrated picture of *C. maxima* fruit depicting pulp and peel (albedo and flavedo).

Fruits of *C. maxima* cultivar ‘Thong Dee’ (Figure 5) planted under organic farming were obtained from Don Faek, Nakhon Chai Si, Nakhon Pathom, Thailand (13°51′43.5″ N 100°13′21.2″ E) in September 2021. The plant sample was deposited at the Project of Institute Establishment for Sireeruckkhachati Nature Learning Park, Mahidol University, Nakhon Pathom, Thailand and identified by Dr. Sunisa Sangvirotjanapat. The voucher specimens were assigned as PBM 006011–006013.

The albedo part was washed and dried in an oven at 60 °C for 24 h, and then was ground to fine powder using a Philips 600 W series grinder (Philips Electronic Co., Ltd., Jakarta, Indonesia). The powdery sample was kept at −80 °C until extraction. For extraction, the sample was mixed with various concentrations of aqueous ethanol at 1:10 (powder:solvent) ratio. The sample was shaken in a shaking water bath (Memmert GmBh, Eagle, WI, USA) under the conditions shown in Table 1. The supernatant was collected through centrifugation at 4500× *g* for 15 min using a refrigerated centrifuge (Hettich^®^ ROTINA 38R, Andreas Hettich GmbH, Tuttlingen, Germany) and filtered through a 0.45 µm polytetrafluoroethylene membrane syringe filter. The filtrates were kept at −20 °C until analysis.

### 4.3. Determination of Total Flavonoid Contents

The TFCs were determined using a well-established method, as previously reported [38], with modifications as follows. Briefly, the sample (100 µL) was mixed with 5% (*w*/*v*) NaNO_2_ (10 µL) and allowed to incubate for 6 min. To the mixture, 10% (*w*/*v*) AlCl_3_·6H_2_O (20 µL) was added and allowed to incubate for another 5 min. The reaction was stopped by adding 1 M NaOH (70 µL) and the mixture was transferred into a 96-well plate. The TFCs were measured at 510 nm using a SynergyTM HT 96-well UV-visible microplate reader (BioTek Instruments, Inc., Winooski, VT, USA) and Gen 5 data analysis software. The results were expressed as mg QE/g DW through the calibration curve of quercetin in the range 10–1000 µg/mL.

### 4.4. Determination of Total Phenolic Contents

The TPCs were determined using a well-established Folin–Ciocalteu assay, as previously reported [39], with modifications as follows. Briefly, the sample (25 µL) was mixed with 10% (*v*/*v*) Folin–Ciocalteu reagent (50 µL) and incubated for 5 min. To the mixture, 7.5% (*w*/*v*) Na_2_CO_3_ (200 µL) was added, and the reaction was left for 120 min in dark. The TPCs were measured at 765 nm using the microplate reader. The results were expressed as mg GAE/g DW through the calibration curve of gallic acid in the range 10–200 µg/mL.

### 4.5. Determination of Antioxidant Properties

Antioxidant activities were analyzed through DPPH radical scavenging and FRAP and ORAC assays using the well-established protocols without any modifications [39]. Briefly, DPPH radical scavenging assay consisted of DPPH free radical solution and an end-point absorbance at 520 nm, while FRAP assay was employed using FRAP reagent containing Fe^3+^-TPTZ solution and an end-point absorbance at 595 nm. The kinetic ORAC assays utilized AAPH peroxyl radical and fluorescein reagent, with an excitation wavelength of 485 nm and an emission wavelength of 535 nm. The results were expressed as µmol TE/g sample through the calibration curve of Trolox in the ranges 0.01–0.64 mM, 7.8125–250 µM, and 3.125–100 µM for DPPH radical scavenging and FRAP and ORAC assays, respectively.

### 4.6. Determination of Enzyme Inhibitory Activities

The enzyme inhibitory activities of AChE, BChE, BACE-1, lipase, α-glucosidase, and α-amylase were determined according to the well-established protocol, as previously reported [40], and summarized in Table 8.

The visualization of enzyme reactions was performed utilizing the microplate reader. Equation (2) was used to determine the inhibitory activity in enzyme assays.

(2) $$\%\;\mathrm{inhibition}=\left(1-\;\frac{B-b}{A-a}\right)\;\times \;100,$$

where *A* is the initial velocity (V) of the reaction with enzyme and blank solvent (control), *a* is the V of reaction with blank solvent but without enzyme (control blank), *B* is the V of the reaction with enzyme and pomelo peel extract (sample), and *b* is the V of the reaction with the pomelo peel extract but without the enzyme (sample blank).

### 4.7. Determination of Phytochemical Profiles Utilizing Liquid Chromatography–Electrospray Ionization–Tandem Mass Spectrometry (LC–ESI–MS/MS)

Phytochemical analysis was performed using LC–ESI–MS/MS with parameters and validations according to the previous studies without any modification [41]. To prepare the extract powder, the solvent of an aqueous ethanolic extract performed under optimized extraction condition was removed by rotary evaporator. The remaining residue was dried at 37 °C overnight in a hot air oven. The dry extract then underwent either non-acidic hydrolysis or acidic hydrolysis. Under the non-acidic hydrolysis condition, the dry extract (0.5 g) was re-dissolved in 62.5% (*v*/*v*) methanol containing 0.5 g *tert*-butylhydroquinone (50 mL) and filtered through a 0.22 µm polyethersulfone (PES) syringe filter. For the acidic hydrolysis condition, the powder (0.5 g) was dissolved in formic acid (40 mL) and 62.5% (*v*/*v*) methanol containing 0.5 g *tert*-butylhydroquinone (10 mL) and was shaken in an 80 °C water bath shaker (TW20 series from Julabo GmbH, Seelbach, Germany) for 2 h. The sample was filtered through a 0.22 µm PES syringe filter. The extracts that had undergone either acidic or non-acidic hydrolysis were loaded onto an Accucore RP-MS column (a 2.1 mm × 100 mm, 2.6 µm column from Thermo Fisher Scientific, Bremen, Germany), which was connected to the LC–ESI–MS/MS system consisting of a Dionex Ultimate 3000 series ultra-high-performance liquid chromatograph (UHPLC) system, a diode array detector, a TSQ Quantis Triple Quadrupole mass spectrometer (MS) and a Chromeleon 7 chromatography data system (version 7.2.9.11323, Thermo Fisher Scientific, Bremen, Germany). The mobile phase consisted of acetonitrile (solvent A) and 0.1% (*v*/*v*) formic acid in Milli-Q water (18.2 MΩ·cm resistivity at 25 °C, solvent B), while the flow rate was set at 0.5 mL/min throughout the experiment. A gradient solvent system was set at 10–80% solvent A (time 0.0–8.0 min), 80–10% solvent A (time 8.0–8.1 min), and 10% solvent A (time 8.1–10.0 min).

Authentic standards for LC–ESI–MS/MS analysis consisted of syringic acid (>97.0% T), sinapic acid (>99.0% GC, T), quercetin (>98.0% HPLC, E), naringenin (>93.0% HPLC, T), myricetin (>97.0% HPLC), luteolin (>98.0% HPLC), kaempferol (>97.0% HPLC), hydroxybenzoic acid (>99.0% GC, T), 4–hesperidin (>90.0% HPLC, T), genistein (>98.0% HPLC), ferulic acid (>98.0% GC, T), (−)-epigallocatechin gallate (>98.0% HPLC), 3,4-dihydroxybenzoic acid (≥97% T), *p*-coumaric acid (>98.0% GC, T), cinnamic acid (>98.0% HPLC), chlorogenic acid (>98.0% HPLC, T), caffeic acid (>98.0% HPLC, T), and apigenin (>98.0% HPLC) from Tokyo Chemical Industry (Tokyo, Japan), vanillic acid (≥97% HPLC), rutin (≥94% HPLC), rosmarinic acid (≥98% HPLC), and gallic acid (97.5–102.5% T) from Sigma-Aldrich (St. Louis, MO, USA), galangin (≥98.0% HPLC) from Wuhan ChemFaces Biochemical Co., Ltd. (Wuhan, China), and isorhamnetin (≥99.0% HPLC) from Extrasynthese (Genay, France).

### 4.8. Gas Chromatography–Mass Spectrometry (GC–MS) Analysis

GC–MS analysis was performed according to the previous report [9] using an Agilent 7000E triple quadrupole GC coupled with a 5975 inert Mass Selective Detector (MSD) (Agilent Technologies, Palo Alto, CA, USA). The samples (1 µL) were injected under liquid mode by an Automatic Liquid Sampler Agilent 7693A (Agilent Technologies, Palo Alto, CA, USA). The device was equipped with the fused silica capillary column (30 m × 0.25 mm × 0.25 µm DB-WAX, Agilent Technologies, Palo Alto, CA, USA) on high polarity. Helium served as the carrier gas, at a flow rate of 1.2 mL/min. The injector was set at 250 °C. The GC oven temperature was programmed at a constant temperature at 50 °C for 5 min, then the temperature increased at 5 °C/min to 230 °C and was held at this temperature for 60 min. The flame ionization detection (FID) temperature was set at 250 °C, and MSD was operated in an electron impact (EI) mode at 70 eV. Identification of the eluted compounds was reached by spectral comparison with NIST library (NIST 17, Software Version 2.3 g, National Institute of Standards and Technology, Gaithersburg, MD, USA).

### 4.9. Bacterial Reverse Mutation Test (Ames Test)

The *C. maxima* albedo extract (10–2000 µg/plate) was tested for its mutagenicity potential using five strains of *Salmonella typhimurium* including TA98, TA100, TA102, TA1535, and TA1537 in both the presence and absence of metabolic activation, as suggested by the OECD guideline for testing of chemicals No. 471 ‘Bacterial Reverse Mutation Test’ [8]. Briefly, each bacterial strain was grown in a nutrient broth (12 mL) at 37 °C for 16 h. Then, 100 µL of the culture (optical density of 0.3–0.4) was added to the test tube containing 50 µL of different doses of extracts and 500 µL of phosphate-buffered saline or 500 µL of S9 mixture and then pre-incubated at 37 °C for 20 min. After the pre-incubation, the mixture was added to 2 mL of top agar containing 0.5 mM l-histidine and 0.5 mM D-biotin. The mixture was agitated and poured evenly onto the minimal agar plate and left to solidify. All experiments were performed in triplicate and incubated at 37 °C for 48 h. After that, the number of revertant colonies per plate was counted. For experiments without metabolic activation, positive controls were 4-NQO (0.2 µg/plate for TA98), NaN_3_ (2.5 µg/plate for TA100 and 0.5 µg/plate for TA1535), MMC (0.5 µg/plate for TA102), and 9-AA (50 µg/plate for TA1537). For experiment with metabolic activation, a positive control, 2-AA, was used. The MR was calculated from the average of the revertant number divided by the average of negative control revertant number [42].

### 4.10. Statistical Analysis

The experimental design, regression, and graphical analysis of the generated data were performed using the software Design-Expert (Stat-Ease Inc., Minneapolis, MN, USA). All experiments were performed in triplicate (*n* = 3) and reported as mean ± standard deviation (SD). The one-way analysis of variance (ANOVA) and Duncan’s multiple comparison were used to examine the difference between samples in experiments. *p* < 0.05 was considered as a significant difference.

## 5. Conclusions

This study showed that *C. maxima* albedo, as food waste, was optimally extracted using RSM to obtain high phytochemical content, especially hesperidin and naringenin. The extraction technique was very straightforward and environmentally friendly, as a green solvent was utilized. The optimum condition for extraction used 50% (*v*/*v*) aqueous ethanol at 30 °C for 4 h. The obtained extract was rich in hesperidin and naringenin and exhibited health benefits, particularly anti-AD through BACE-1 inhibition. The extract was also genome-safe because it did not induce DNA mutations. This study may be used as a sustainable approach to reducing food waste and adding economic value to *C. maxima* albedo by implementation in the functional food sector.

## Figures and Tables

**Table 1:** Coded and uncoded independent variables (ethanol concentration, extraction temperature, and extraction time) and dependent variables (total phenolic contents (TPCs) and total flavonoid contents (TFCs)) derived from the Box–Behnken design (BBD) for *C. maxima* albedo extraction.

Run	X_1_: Ethanol (% *v*/*v*)		X_2_: Temperature (°C)		X_3_: Time (h)		TPCs (mg GAE/g DW)	TFCs (mg QE/g DW)
	Coded	Uncoded	Coded	Uncoded	Coded	Uncoded	Experimental	Experimental
1	−1	50	1	70	0	4	13.02 ± 0.03 ^f^	3.26 ± 0.01 ^c^
2	−1	50	0	50	1	6	1.84 ± 0.17 ^k^	3.07 ± 0.01 ^d^
3	0	70	1	70	−1	2	20.35 ± 0.01 ^c^	1.76 ± 0.02 ^i^
4	0	70	1	70	1	6	20.59 ± 0.04 ^b^	1.51 ± 0.01 ^j^
5	0	70	0	50	0	4	11.39 ± 0.01 ^i^	1.43 ± 0.02 ^k^
6	0	70	0	50	0	4	11.34 ± 0.00 ^i^	1.40 ± 0.01 ^k^
7	1	90	1	70	0	4	12.16 ± 0.01 ^h^	2.87 ± 0.02 ^e^
8	0	70	0	50	0	4	11.38 ± 0.03 ^i^	1.46 ± 0.02 ^k^
9	−1	50	0	50	−1	2	4.47 ± 0.029 ^j^	3.71 ± 0.03 ^b^
10	1	90	−1	30	0	4	15.46 ± 0.03 ^e^	2.08 ± 0.05 ^h^
11	0	70	−1	30	1	6	11.36 ± 0.01 ^i^	2.34 ± 0.02 ^g^
12	1	90	0	50	1	6	11.34 ± 0.02 ^i^	1.45 ± 0.04 ^k^
13	−1	50	−1	30	0	4	15.79 ± 0.03 ^d^	4.50 ± 0.01 ^a^
14	1	90	0	50	−1	2	21.88 ± 0.04 ^a^	1.48 ± 0.02 ^jk^
15	0	70	−1	30	−1	2	12.92 ± 0.03 ^g^	2.55 ± 0.05 ^f^

Experimental data are shown as mean ± standard deviation (SD) of triplicate experiments (*n* = 3). Superscript letters in each column indicate significantly different TPCs or TFCs in each run condition from BBD at *p* < 0.05 calculated by one-way analysis of variance (ANOVA) and Duncan’s multiple comparison test. GAE, gallic acid equivalent; QE, quercetin equivalent; DW, dry weight.

**Table 2:** Coefficient of determination, regression coefficients, and *p*-value of the second-order polynomial models for total phenolic contents (TPCs) and total flavonoid contents (TFCs).

Source	TPCs			TFCs		
	Coefficient	*p*-Value	Significance	Coefficient	*p*-Value	Significance
Model	11.38	0.4440	ns	1.30	0.0030	**
X_1_	0.7000	0.7033		−0.8347	0.0005	**
X_2_	3.24	0.1085		−0.2574	0.0590	
X_3_	−1.16	0.5326		−0.1415	0.2386	
X_1_X_2_	−0.1325	0.9591		0.5069	0.0195	*
X_1_X_3_	−0.7980	0.7584		0.1534	0.3520	
X_2_X_3_	−1.92	0.4669		−0.0102	0.9482	
$X_{1}^{2}$	5.17	0.0711		1.13	0.0008	***
$X_{2}^{2}$	−2.44	0.3494		0.7478	0.0049	**
$X_{3}^{2}$	1.00	0.6919		−0.0038	0.9817	
R^2^	0.5923			0.9686		
R^2^ adjusted	0.0703			0.9122		
Lack of fit	1.59	<0.0001	****		0.4086	ns

Statistical analyses were determined by one-way analysis of variance (ANOVA). *, *p* < 0.05; **, *p* < 0.01; ***, *p* < 0.001; ****, *p* < 0.0001; ns, not significant; X_1_, ethanol concentration; X_2_, extraction temperature; X_3_, extraction time.

**Table 3:** Phenolic profiles of *C. maxima* albedo extract prepared under acidic and non-acidic hydrolysis.

Phenolics	Amount (µg/g DW)
**Non-acid hydrolysis**	
Hesperidin	161.03 ± 5.03
**Acid hydrolysis**	
Apigenin	41.96 ± 1.29
Naringenin	3430.41 ± 135.02
Sinapic acid	142.32 ± 11.92

All data are expressed as mean ± standard deviation (SD) of triplicate experiments (*n* = 3).

**Table 4:** The volatile compounds in *C. maxima* albedo detected using gas chromatography–mass spectrometry (GC–MS) analysis.

Peak Number with Possibility Score of More Than 50%	Retention Time (min)	Name	Formula	Area (%)
1	8.683	Limonene	C_10_H_16_	4.05
2	30.060	Phosphonic acid, (phydroxyphenyl)-	C_6_H_7_O_4_P	4.28
3	37.934	Catecholborane	C_6_H_5_BO_2_	9.26

Only the peaks with a possible score of more than 50% are reported (spectra matching with the National Institute of Standards and Technology (NIST) library).

**Table 5:** Antioxidant activities and enzyme inhibitory activities of *C. maxima* albedo extracted under optimized extraction condition.

Types	Analyses	Values
Antioxidant activities	DPPH (µmol TE/g DW)	15.35 ± 0.47
	FRAP (µmol TE/g DW)	9.36 ± 0.06
	ORAC (µmol TE/g DW)	675.34 ± 22.16
Enzyme inhibitions	AChE (% inhibition) ^#^	14.57 ± 1.17
	BChE (% inhibition) ^#^	26.76 ± 1.17
	BACE-1 (% inhibition) ^$^	54.61 ± 5.08
	Lipase (% inhibition) ^#^	20.89 ± 0.35
	α-Glucosidase (% inhibition) ^#^	45.40 ± 1.73
	α-Amylase (% inhibition) ^#^	35.38 ± 2.24

All data are shown as mean ± standard deviation (SD) of triplicate experiments (*n* = 3). DPPH, 2,2–diphenyl–1–picrylhydrazyl; TE, Trolox equivalent; DW, dry weight; FRAP, ferric ion reducing antioxidant power; ORAC, oxygen radical absorbance capacity; AChE, acetylcholinesterase; BChE, butyrylcholinesterase; BACE-1, β-secretase. ^#^ final concentration of *C. maxima* peel extracts at 25 mg/mL; ^$^ final concentration of *C. maxima* peel extracts at 8 mg/mL.

**Table 6:** Mutagenicity effects of *C. maxima* albedo extract on five *S. typhimurium* strains without rat liver S9 extract (−S9).

Doses (µg/Plate)	TA98		TA100		TA102		TA1535		TA1537	
	Revertant Colonies	MR	Revertant Colonies	MR	Revertant Colonies	MR	Revertant Colonies	MR	Revertant Colonies	MR
Neg	87.67 ± 4.61	1.00 (−)	71.17 ± 2.73	1.00 (−)	373.33 ± 4.46	1.00 (−)	9.67 ± 1.11	1.00 (−)	8.67 ± 0.94	1.00 (−)
10	85.00 ± 3.00	0.97 (−)	69.50 ± 3.15	0.98 (−)	374.50 ± 6.80	1.00 (−)	11.00 ± 1.15	1.14 (−)	9.17 ± 0.37	1.06 (−)
100	88.33 ± 4.85	1.01 (−)	71.50 ± 2.99	1.00 (−)	376.50 ± 4.79	1.01 (−)	10.33 ± 0.94	1.07 (−)	9.33 ± 0.75	1.08 (−)
500	83.67 ± 3.14	0.95 (−)	73.17 ± 5.34	1.03 (−)	374.67 ± 4.50	1.00 (−)	10.00 ± 1.00	1.03 (−)	8.67 ± 1.37	1.00 (−)
1000	86.17 ± 2.41	0.98 (−)	71.33 ± 2.36	1.00 (−)	373.67 ± 4.03	1.00 (−)	9.83 ± 1.07	1.02 (−)	9.17 ± 1.34	1.06 (−)
2000	85.50 ± 4.19	0.98 (−)	69.00 ± 3.27	0.97 (−)	374.00 ± 7.85	1.00 (−)	10.67 ± 1.11	1.10 (−)	10.00 ± 0.82	1.15 (−)
4-NQO	1047.33 ± 32.20	11.95 (+)								
NaN_3_			1146.67 ± 22.82	16.11 (+)			271.17 ± 5.81	28.05 (+)		
MMC					1108.67 ± 19.92	2.97 (+)				
9-AA									776.67 ± 33.02	89.62 (+)

All data are shown as mean ± standard deviation (SD) of triplicate experiments (*n* = 3). Negative control (Neg) is dimethyl sulfoxide (DMSO) used as a solvent control. (−) indicates the mutagenicity ratio (MR) is ≤1, and (+) indicates the mutagenicity ratio (MR) is ≥2. MR, mutagenicity ratio; positive control: 4NQO, 4-nitroquinoline-1-oxide; NaN_3,_ sodium azide; MMC, mitomycin C; 9-AA, 9-aminoacridine.

**Table 7:** Mutagenicity effects of *C. maxima* albedo extract on five *S. typhimurium* strains with rat liver S9 extract (+S9).

Doses (µg/plate)	TA98		TA100		TA102		TA1535		TA1537	
	Revertant Colonies	MR	Revertant Colonies	MR	Revertant Colonies	MR	Revertant Colonies	MR	Revertant Colonies	MR
Neg	89.83 ± 3.72	1.00 (−)	85.83 ± 3.29	1.00 (−)	375.00 ± 4.00	1.00 (−)	10.00 ± 1.15	1.00 (−)	9.50 ± 0.96	1.00 (−)
10	92.00 ± 3.27	1.02 (−)	84.50 ± 2.57	0.98 (−)	380.33 ± 5.65	1.01 (−)	11.33 ± 1.11	1.13 (−)	9.67 ± 0.94	1.02 (−)
100	94.17 ± 3.58	1.05 (−)	82.00 ± 2.38	0.96 (−)	374.33 ± 4.35	1.00 (−)	10.33 ± 1.11	1.03 (−)	9.33 ± 0.94	0.98 (−)
500	95.17 ± 2.54	1.06 (−)	81.50 ± 3.64	0.95 (−)	383.50 ± 5.62	1.02 (−)	10.67 ± 1.37	1.07 (−)	10.67 ± 1.25	1.12 (−)
1000	90.17 ± 1.46	1.00 (−)	81.83 ± 4.41	0.95 (−)	374.00 ± 3.70	1.00 (−)	10.83 ± 1.57	1.08 (−)	11.17 ± 0.90	1.18 (−)
2000	90.33 ± 3.54	1.01 (−)	82.83 ± 6.79	0.97 (−)	374.83 ± 5.01	1.00 (−)	11.17 ± 0.69	1.12 (−)	10.83 ± 1.07	1.14 (−)
2-AA	1134.67 ± 53.80	12.63 (+)	1006.67 ± 40.24	11.73 (+)	1118.00 ± 23.20	2.98 (+)	369.83 ± 8.39	36.98 (+)	200.83 ± 6.44	21.14 (+)

All data are shown as mean ± standard deviation (SD) of triplicate experiments (*n* = 3). Negative control (Neg) is dimethyl sulfoxide (DMSO) used as a solvent control. (−) indicates the mutagenicity ratio (MR) is ≤1, and (+) indicates the mutagenicity ratio (MR) is ≥2. MR, mutagenicity ratio; 2-AA, 2-aminoanthracen.

**Table 8:** The enzyme assay components.

Assay	Assay Components				
	Enzyme	Substrate	Indicator	Extract	Detection Wavelength
AChE	100 μL of 0.25 µg/mL AChE ^1^	50 μL of 0.32 mM ACh	10 µL of 16 mM DTNB	40 µL	412 nm
BChE	100 μL of 1.5 µg/mL BChE ^2^	50 μL of 0.4 mM BCh			
Lipase	100 µL of 5 µg/mL lipase ^3^	50 μL of 0.2 mM DMPTB			
BACE-1	BACE-1 FRET assay kit (Sigma-Aldrich, St. Louis, MO, USA) following manufacturer’s recommendations				λ_ex_ = 320 nm λ_em_ = 405 nm
α-Amylase	100 µL of 50 mg/mL α-amylase ^4^	50 µL of 30 mM pNPM		50 µL	405 nm
α-Glucosidase	100 µL of 0.1 U/mL α-glucosidase ^5^	50 µL of 2 mM pNPG + 160 µL KPB (pH 7)		50 µL	

^1^*Electrophorus electricus* AChE (1000 units/mg); ^2^ equine serum BChE (≥10 units/mg); ^3^ *Candida rugosa* lipase (type VII, ≥700 unit/mg); ^4^ porcine pancreatic α-amylase (type VII, ≥10 unit/mg); ^5^ *Saccharomyces cerevisiae* α-glucosidase (type I, ≥10 U/mg protein). AChE, acetycholinesterase; Ach, acetylthiocholine; DTNB, 5,5′-dithiobis(2-nitrobenzoic acid); BChE, butyrylcholinesterase; BCh, butyrylthiocholine; DMPTB, 2,3-dimercapto-1-propanol tributyrate; BACE-1, β-secretase; FRET, fluorescence resonance energy transfer; pNPM, *p*-nitrophenyl-α-d-maltohexaoside; pNPG, *p*-nitrophenyl-α-d-glucopyranoside; KPB, potassium phosphate buffer.

## Data Availability

Data are contained within this article.

## Sample Availability

Plant samples are available from the authors upon reasonable request.
